# ESCRT-III-associated proteins and spastin inhibit protrudin-dependent polarised membrane traffic

**DOI:** 10.1007/s00018-019-03313-z

**Published:** 2019-10-05

**Authors:** James W. Connell, Rachel J. Allison, Catherine E. Rodger, Guy Pearson, Eliska Zlamalova, Evan Reid

**Affiliations:** 1 Department of Medical Genetics and Cambridge Institute for Medical Research, The Keith Peters Building, Cambridge Biomedical Campus, University of Cambridge, Cambridge, CB2 0XY UK; 2 Present Address: Alzheimer’s Research, Cambridge, UK

**Keywords:** Protrusion formation, AAA ATPase, Axonopathy, Bone morphogenetic protein signalling, Microtubule modification

## Abstract

Mutations in the gene encoding the microtubule severing ATPase spastin are the most frequent cause of hereditary spastic paraplegia, a genetic condition characterised by length-dependent axonal degeneration. Here, we show that HeLa cells lacking spastin and embryonic fibroblasts from a spastin knock-in mouse model become highly polarised and develop cellular protrusions. In HeLa cells, this phenotype was rescued by wild-type spastin, but not by forms unable to sever microtubules or interact with endosomal ESCRT-III proteins. Cells lacking the spastin-interacting ESCRT-III-associated proteins IST1 or CHMP1B also developed protrusions. The protrusion phenotype required protrudin, a RAB-interacting protein that interacts with spastin and localises to ER–endosome contact sites, where it promotes KIF5-dependent endosomal motility to protrusions. Consistent with this, the protrusion phenotype in cells lacking spastin also required KIF5. Lack or mutation of spastin resulted in functional consequences for receptor traffic of a pathway implicated in HSP, as Bone Morphogenetic Protein receptor distribution became polarised. Our results, therefore, identify a novel role for ESCRT-III proteins and spastin in regulating polarised membrane traffic.

**Electronic supplementary material:**

The online version of this article (10.1007/s00018-019-03313-z) contains supplementary material, which is available to authorized users.

## Introduction

The hereditary spastic paraplegias (HSPs) are genetic conditions in which there is distal degeneration of the longest axons of the corticospinal tract, the main central nervous system pathway that connects the motor cerebral cortex to the spinal cord [1]. This causes HSP patients to develop progressive and disabling lower limb weakness and stiffness. Mutations in more than 70 genes that cause HSP have been identified, and analysis of the proteins encoded by these genes has revealed that they function in a relatively small number of cellular processes, prominent amongst which is membrane traffic [2, 3].

The function of spastin is particularly important in understanding the pathogenesis of HSP, as mutations in SPAST/SPG4, the gene encoding it, are by far the most common cause of the condition in northern Europe and North America [4]. Spastin is a microtubule severing ATPase enzyme, which oligomerises as a hexamer before using the energy provided by ATP hydrolysis to create internal breaks or nanoscale damage (“nicks”) in microtubules [5, 6]. The SPAST mutational spectrum in HSP includes nonsense, frameshift, splicing and exon/whole gene deletion mutations, indicating that in most cases the molecular pathological mechanism is haploinsufficiency [7–11]. Missense mutations affecting the ATPase domain are also commonly found. These have been reported to affect ATP binding, ATP hydrolysis or oligomerisation of the protein, and so these mutations also likely cause loss of spastin function [12], although it is possible that mutations affecting ATP binding or hydrolysis could exert a dominant negative effect on the spastin hexamer, perhaps blocking the ATPase cycle at a point where spastin is bound to microtubules [13].

We showed recently that spastin participates in molecular machinery at ER–endosome contacts that drives fission of endosomal tubules. These tubules arise from endosomal compartments and are involved in recycling and retrieval of receptors, such as the transferrin receptor (TfnR) or mannose 6-phosphate receptors (M6PRs), back to the plasma membrane (directly, or indirectly via the perinuclear recycling compartment) or to the Golgi apparatus [14]. Formation, breakage and transport of these tubules occur along microtubules and require microtubule-dependent motors, such as kinesins (for plus-end-directed transport) and dynein (for minus-end-directed transport) [15, 16]. Proteins that are not sorted away from endosomes via these and other pathways are trafficked to the late endosomal/lysosomal pathway for degradation [14]. Spastin localises to contacts between the ER and endosome that are sites of endosomal tubule fission. These contacts involve an interaction between an ER-localised isoform of spastin (M1-spastin) and the endosomal protein IST1, an atypical member of the ESCRT-III (endosomal sorting complex required for transport-III) complex [15, 17]. Spastin is required for efficient tubule fission, so that TfnRs and M6PRs are retained in the endo-lysosomal pathway in cells lacking spastin. Spastin requires its ATPase and ESCRT-interacting functions to promote endosomal tubule fission [15, 17].

The location of spastin at ER–endosome contacts parallels that of another spastin interactor, protrudin/ZFYVE27. Protrudin was first characterised as a driver of polarised membrane traffic. HeLa cells expressing protrudin develop long microtubule-based cellular protrusions, while protrudin promoted neurite extension in rat hippocampal neurons and PC12 cells. In PC12 cells, NGF treatment resulted in movement of protrudin from its predominant steady-state localization in the ER to what was described as a RAB11-positive endosomal compartment at the tip of neurite extensions [18]; however, recent work suggests that this apparent redistribution to endosomes actually reflects increased contact between this organelle and ER-localised protrudin [19].

Two mechanisms have been proposed for how protrudin drives cellular protrusion formation. Protrudin contains a RAB-binding domain (RBD) that preferentially binds to RAB11-GDP, which it locks in this inactive state. This binding is enhanced by ERK-driven protrudin phosphorylation that occurs downstream of NGF treatment. Thus, it has been proposed that protrudin inhibits RAB11-dependent generalised (i.e., non-polarised) endosomal recycling [18]. In addition, protrudin has functional and physical interactions with kinesin motor KIF5 proteins. Protrudin interacts with KIF5A (a neuron-specific kinesin) in brain tissue, protrudin colocalises with KIF5 and RAB11 in the cell body and protrusions of HeLa cells expressing protrudin, expression of KIF5A in HeLa cells promotes protrusion formation, while depletion of KIF5B (the endogenous KIF5 present in HeLa cells) reduces the protrusion phenotype seen on protrudin expression [20, 21]. More recently, protrudin has been shown to bind late endosomal RAB7 at ER–endosome contact points, where it promotes transfer of KIF5 to the endosomal motor protein adaptor FYCO1, to promote polarised traffic of late endosomes [19]. Considered together, these observations suggest a model where increased association between protrudin and endosomal RAB proteins at ER–endosome contact sites drives polarised membrane traffic by switching off RAB11-GTP-dependent generalised endosomal recycling, and promoting KIF5-dependent polarised traffic into protrusions.

In light of its interaction with protrudin, we hypothesised that spastin might also function in polarised membrane traffic. Here, we show that primary cells from a spastin mouse knocked in for a disease-associated ATPase mutation and HeLa cells lacking spastin become highly polarised, with the development of long cellular protrusions that require protrudin and KIF5 for their development. This polarisation phenotype could be rescued by wild-type spastin, but not by a form unable to bind to ESCRT-III. Consistent with this, cells lacking the ESCRT-III proteins that bind spastin also developed protrusions. The polarisation phenotype had functional consequences for receptor traffic, as HeLa cells lacking spastin and cells from the knock-in mouse had altered plasma membrane distribution of Bone Morphogenetic Protein signalling receptors, a pathway implicated in HSP. Thus, considered with our previous work showing that spastin promotes generalised endosomal recycling, our results indicate that microtubule modelling by spastin regulates the balance between generalised and protrudin-dependent polarised endosomal membrane traffic.

## Materials and methods

### Antibodies

Rabbit polyclonal anti-spastin (86-340) was produced as previously described [23]. Mouse monoclonal anti-spastin (6C6) and mouse monoclonal anti-Acetylated tubulin (6-11B-1) were obtained from Sigma-Aldrich. Mouse monoclonal anti-myc (4A6) was obtained from Millipore. Rabbit polyclonal anti-myc tag (ab9106), rabbit anti-GFP (ab6556) and rat anti-alpha-tubulin-tyrosinated clone YL12/2 (ab6160) were obtained from Abcam. Rat anti-RFP (5F8) was obtained from Chromotek. Goat anti-BMPR2 (EB08820) and goat anti-Kinesin-1/KIF5 (EB05492) were obtained from Everest Biotech. Rabbit polyclonal anti-ZFYVE27 (12680-1AP) and anti-IST1 (51002-1-AP) were obtained from Proteintech. Mouse monoclonal anti-TfnR (13-6800) was obtained from Invitrogen. Rabbit polyclonal anti-GAPDH (2118) was obtained from Cell Signaling Technology. Rabbit polyclonal anti-CHMP1B was produced as previously described [15]. Peroxidase conjugated secondary antibodies for western blotting were obtained from Sigma-Aldrich. Alexafluor-labelled secondary antibodies for immunofluorescence were obtained from Molecular Probes.

### Constructs and generation of stable cell lines

pLXIN-myc-Spastin M1 wild-type (wt) and M87 wt, K388R and F124D constructs for generation of stable cell lines were made as previously described [15]. Briefly M87 or M87A-M1 Spastin was cloned into pIRESneo2 (Clontech) followed by insertion of a myc tag (mutagenesis of the M87 residue ensures that no M87 transcript is produced from the M1 construct). Myc-spastin was further cloned into SalI-BamHI sites in pLXIN. pLXIN-myc-M87-Spastin-K388R and F124D mutants were then generated and all constructs were made resistant to spastin siRNA 1 and 3 by introducing two mutations into each of the relevant sequences by site-directed mutagenesis. Myc-BMRP2 stable cell lines were generated by PCR of the myc-BMRP2 construct described in [22] and cloning the myc-BMPR2 fragment into pLXIN, followed by retroviral transduction of HeLa cells.

### Cell culture, transfection and siRNAs

HelaM and MRC5 cells were maintained as previously described [23]. HeLaM cells stably expressing spastin constructs were additionally cultured in the presence of 500 μg/ml Geneticin (Invitrogen). Mouse embryonic fibroblasts (MEFs) were obtained from E14 mouse embryos and cultured in DMEM containing 10% foetal calf serum, 1% penicillin–streptomycin (Invitrogen), 1% l-glutamine (Invitrogen), 1% MEM non-essential amino acids (Invitrogen) and 0.1% 2-mercaptoethanol (Invitrogen). For transient transfections of plasmid DNA, HeLaM cells were transfected using Effectine (Qiagen) following the manufacturer’s protocol, and incubated for 24 h before analysis. For siRNA transfection, cells were transfected with the relevant siRNAs, using Oligofectamine transfection reagent (Invitrogen), according to a 5-day protocol modified from [24]. Briefly, HeLaM or MRC5 cells were plated into a well of a 6-well plate and transfected with siRNA after 24 h. Cells were harvested 96 h later. The efficiency of siRNA knock-down was verified by immunoblotting cell lysates and/or by immunofluorescence microscopy of fixed cells, with an antibody against the relevant protein, or in the case of some protrudin depletion experiments (performed when an endogenous protrudin antibody was not available), by carrying out parallel knock-down experiments in cells transiently expressing GFP-protrudin and validating protein knock-down using an anti-GFP antibody for immunoblotting. The following siRNAs were obtained from Dharmacon and used at the indicated concentrations:

- Spastin (5 nM), siRNA1: 5′-GAACUUCAACCUUCUAUAA, siRNA3: 5′-UAUAAGUGCUGCAAGUUUA.
- Protrudin (20 nm), siRNA3: 5′-GUAACCAGACCUUGAGCAA; siRNA4: 5′-ACAAGAGGCUGGAGAUCUA.
- KIF5B (10 nm) siRNA1: 5′-GCAGUCAGGUCAAAGAAUA; siRNA custom2: 5′-UGAAUUGCUUAGUGAUGAAUUUU.
- IST1 (25 nM), siRNA1: 5′-CCAAGUAUAGCAAGGAAUA-3′ (D-020977-01); siRNA3, 5′-GCAAAUACGCCUUUCUCAU-3′ (D-020977-03).
- CHMP1B (10 nM), siRNA: 5′-GAAGAUUUCUGCUUUGAUGUU-3′ (D-004698-01); siRNA6: 5′-GUCGAUGGCUGGUGUGGUU-3′ (J-004698-06); siRNA7: 5′-CCUUCGGGAUCAAGUGUGA-3′ (J-004698-07).

### Immunofluorescence microscopy of HeLa cells and MEFs

For immunofluorescence co-localisation cells were either fixed at room temperature in 3.7% (v/v) formaldehyde in phosphate-buffered saline (PBS) and permeabilised in PBS containing 0.1% (v/v) Triton-X100 (Sigma) or fixed and permeabilised for 5 min with ice-cold Methanol (Fisher Scientific). Where pre-fixation extraction of cytosolic protein was required, this was carried out using a saponin-based extraction buffer as previously described [25]. Fixed cells on coverslips were blocked in 5% foetal calf serum in PBS for 1 h and incubated with primary antibodies and Alexa Fluor^®^ conjugated secondary antibodies in blocking buffer and mounted onto glass slides using Prolong^®^ Gold antifade reagent with DAPI (Molecular Probes). Cells were visualised using a Zeiss LSM880 confocal microscope using a 63 × oil immersion objective and images captured using Zen software. To quantify the degree of co-localisation between individual pixels in different fluorescent channels, a Pearson’s correlation coefficient with Costes automatic thresholding was calculated using Volocity Software (Quorum Technologies) for individual cells in each image. Images of SNX1 immunofluorescence (i.e., for endosomal tubule quantification) were obtained with a Zeiss AxioImager Z2 Motorized Upright Microscope (63 × NA 1.40 oil immersion objective, room temperature, Zeiss Axiocam 506) and deconvolved with Huygens Professional version 18.04 (Scientific Volume Imaging, The Netherlands, http://svi.nl). Images were subsequently processed using Adobe Photoshop and presented using Adobe Illustrator.

### Protrusion quantification in HeLa Cells and MEFS

HelaM cells were plated at low density (1 × 10^4^) onto poly-d-lysine (Sigma-Aldrich)-coated coverslips in six-well plates. 24 h later, cells were transfected with spastin siRNA with or without rescue siRNA oligonucleotides and incubated for a further 96 h. Cells were then fixed and permeabilised in ice-cold methanol and labelled with anti-acetylated tubulin primary antibodies and Alexa Fluor^®^ conjugated secondary antibodies. Coverslips were mounted onto glass slides using Prolong^®^ Gold antifade reagent with DAPI (Molecular Probes) and cells were visualised using a Zeiss Axioimager Z2 Wide-field upright microscope with a motorised stage and 40 × oil immersion objective. Some images were deconvolved as above. Stitched images of 5 × 5 individual tiles were automatically captured using Zen Pro analysis software and cells in 25 automatically captured fields were analysed per coverslip. Protrusions were defined as cell extensions which were acetylated tubulin positive with a length greater than 2 × the widest diameter of the nucleus, a stringent criterion compared to previous publications that have used a process length greater than 1 × the nuclear diameter [26]. In cells with more than one protrusion, the longest protrusion only was counted. The length of each protrusion was manually measured using the spline curve tool in Zen from the edge of the nucleus to the longest point away from the nucleus identified by acetylated tubulin immunofluorescence. The total number of cells in 25 fields per coverslip was then automatically counted using a nucleus detection protocol or using cellular detection with a whole cell stain (Thermo Scientific) in Zen and the percentage of total cells with protrusions was calculated. For each independent experiment, between 300 and 1000 cells were analysed per siRNA and at least three independent experiments were carried out. For quantification of protrusions in MEFs, the same protocol and measurement parameters were used and in addition protrusions were only counted if the diameter of the half way point along the length of each protrusion was equal to or less than 10 µm.

### Protrusion rescue experiments in stable cell lines

For protrusion rescue experiments in myc-spastin stable cell lines, Zen Pro analysis software was first used to set a fluorescence intensity threshold in the fluorescent channel of the myc tag to identify the total number of stably transfected cells in 25 fields. Protrusions were then counted in all cells identified as stably transfected and the number of stable expressing cells with protrusions was expressed as a percentage of the total.

### Western blotting

Cells were washed twice with PBS and lysed in ice-cold lysis buffer (25 mM HEPES pH 7.4, 1 mM EDTA, 150 mM NaCl, 0.5% TX100, protease inhibitors). Lysate was centrifuged at 13,000 rpm for 15 min and protein concentration in the supernatant was quantified using BCA protein assay (Pierce). 30 µg of protein was resolved using SDS-PAGE and protein gels were blotted onto Nitrocellulose membrane and blocked in 5% non-fat dried milk in TBS-Tween. Membrane was incubated with primary antibodies and HRP-conjugated secondary antibodies in blocking buffer and HRP-signal detected with SuperSignal ^®^ West Pico Chemiluminescent Substrate using X-Ray film (Fuji Medical).

### Statistical analysis

Statistical analysis was performed using unpaired two-tailed Student’s *t* tests or one-way ANOVA with Dunnett’s or Tukey’s post hoc tests for multiple comparisons, using GraphPad Prism 5.01 for Windows (GraphPad Software, San Diego) statistical software. Bars in all histograms represent mean ± standard error of the mean; bars in dot plot represent mean ± standard deviation.

### Animals

Mice were maintained in accordance with UK and European Union regulations. Animal work for this study was approved by the University of Cambridge Ethical Review Committee and was performed under project licenses (80/2304 and 70/7888) granted by the UK Home Office under the Animals (Scientific Procedures) Act 1986. The University of Cambridge is a designated establishment for breeding and scientific procedures under the Animals (Scientific Procedures) Act 1986. Spastin knock-in mice were housed, maintained on a C57BL/6J background and bred to generate embryos by heterozygous breeding as previously described [27].

## Results

### Embryonic fibroblasts from a spastin ATPase-defective knock-in mouse model develop protrusions

We began by examining the morphology of murine embryonic fibroblasts (MEFs) derived from a spastin mouse model (spastin^N386K^) knocked in for an HSP-associated mutation that renders the protein ATPase deficient and unable to sever microtubules. These mice express mutant spastin at endogenous levels and so results are physiologically relevant. In addition, homozygous mutant mice develop a gait phenotype consistent with human HSP and cultured primary neurons from these mice show pathological axonal swellings; thus, these mice appear to be an excellent HSP disease model [17, 27].

**Fig. 1:**
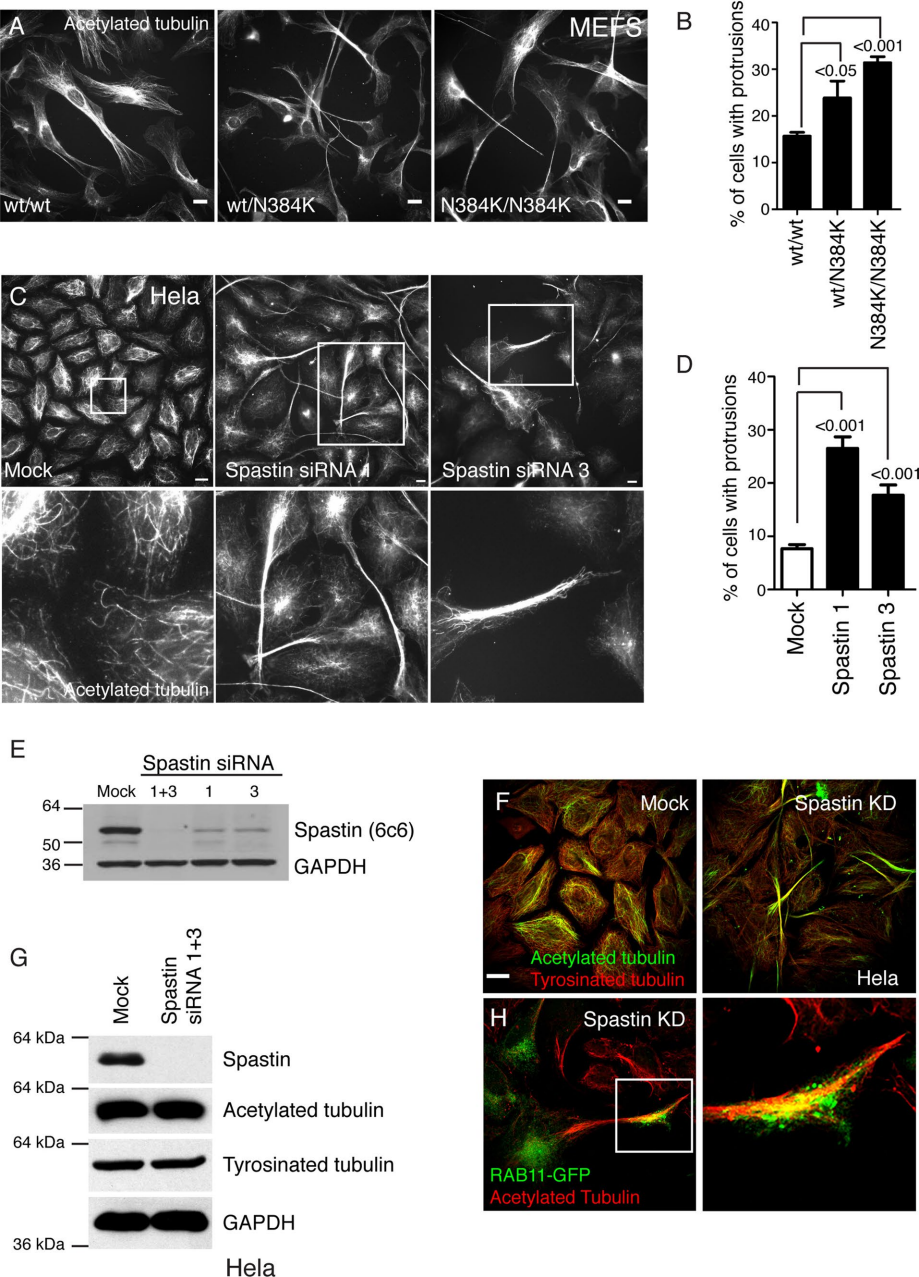
Functional spastin is required to prevent protrusion formation. **a** MEFs from mice with the genotypes indicated were fixed and labelled for acetylated tubulin, and the percentage of cells with protrusions plotted in **b**. *n* = at least 5 biological repeats for each genotype. **c** HeLa cells were mock-transfected, or transfected with the siRNAs indicated, then labelled for acetylated tubulin. The lower panels show higher magnification views of the boxed areas in the upper panels. The percentage of cells with protrusions is quantified in **d**, *n* = 6 biological repeats. **e** Spastin depletion in these experiments was validated by immunoblotting. **f** HeLa cells were mock-transfected or subject to spastin knock-down (KD) and labelled with antibodies to tyrosinated or acetylated tubulin. **g** Mock-transfected HeLa cells or HeLa cells transfected with the siRNAs indicated were lysed and immunoblotted for the antibodies shown. **h** HeLa cells depleted for spastin were transfected for RAB11-GFP and labelled for acetylated tubulin. RAB11 accumulates at the tip of protrusions. The right panel shows a magnified view of the boxed area in the left-hand panel. In **e** and **g**, GAPDH blotting serves to validate equal protein loading. *P* values were generated by one-way ANOVA with Dunnett’s post hoc test for multiple comparisons, histograms in this and all subsequent figures show mean ± SEM. Scale bars = 20 μm

We found a significant increase in cellular protrusions in MEFs derived from spastin^N384K/N384K^ mice versus controls (Fig. 1a, b). The protrusions were filled with linear arrays of microtubules and were defined as cell extensions which were tubulin positive and with a length greater than 2 × the diameter of the nucleus of the cell from which it arose.

We validated the relevance of these findings to humans using HeLa cells, a line that has already been shown to generate protrudin-dependent protrusions [19]. Using two different siRNA oligonucleotides, we saw a highly significant increase in the proportion of cells with protrusions in cells lacking spastin (Fig. 1c–e). These protrusions were typically many times longer than the diameter of the cell body from which they originated (> 60% were longer than 80 μm, i.e., representing 4 × the typical nuclear diameter) and were filled with a linear array of microtubules (Fig. 1c). The microtubules were labelled strongly with antibodies for tyrosinated tubulin, an indicator of dynamic microtubules that are prone to growth and shrinkage, and especially for acetylated tubulin, an indicator of long-lived structures that are associated with long-range transport (Fig. 1f) [28]. These tubulin modifications in protrusions were not associated with alterations in total cellular content of acetylated or tyrosinated tubulin, as measured by immunoblotting (Fig. 1g). The protrusions were also clearly visible upon cellular labelling with a whole cell stain or with the actin marker phalloidin (Supplementary Figure 1). Markers of endosomes accumulated at protrusion tips, including RAB11 (which was present in every protrusion where it was analysed) (Fig. 1h) and transferrin receptor, as well as SNX1 (a marker of endosomal tubules) and RAB5 and 7 (early and late endosomal markers, respectively; not shown). We also observed protrusion formation when spastin was depleted from MRC5 cells, a human fibroblast line, confirming the relevance of this phenotype to cells from at least two human germ layers (Supplementary Figure 1).

We concluded that spastin inhibits protrusion formation in mice and humans, and that this requires its ATPase activity, and hence its ability to modify microtubules.

### ESCRT interaction as well as ATPase activity is required for spastin to regulate polarised membrane traffic in human cells

To analyse further the functional requirements for spastin to prevent protrusion formation, we carried out siRNA-rescue experiments in HeLa cell lines stably expressing siRNA-resistant forms of human spastin.

**Fig. 2:**
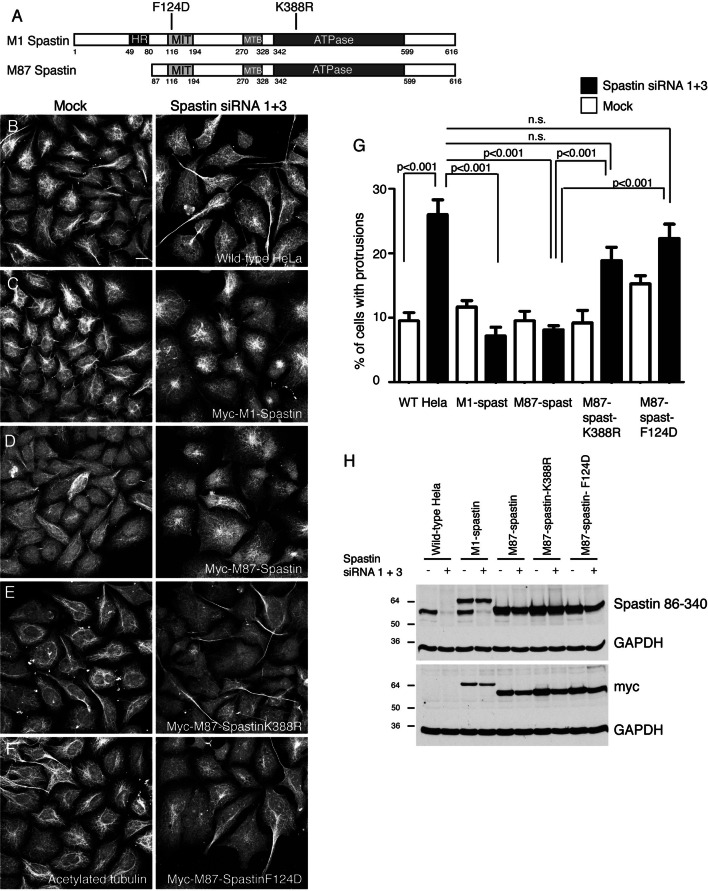

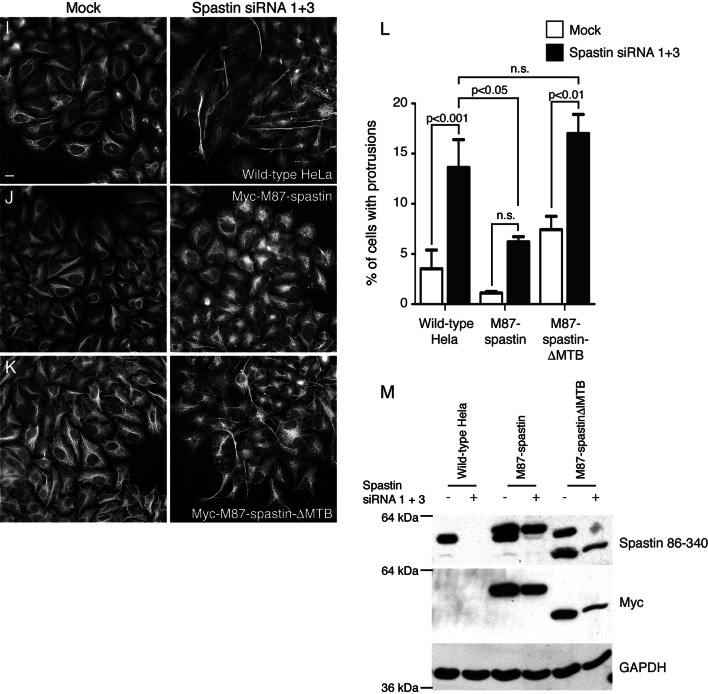
Functional MIT, MTB and ATPase domains are required for spastin to regulate protrusions. **a** Schematic diagram of the M1 and M87 isoforms of spastin. *HR* hydrophobic region (hairpin membrane domain required for ER localisation), *MIT* microtubule interacting and trafficking domain (required for ESCRT-III interaction), *MTB* microtubule binding domain, *ATPase* ATPase domain. The approximate position of the F124D and K388R mutations used in this study is indicated. **b**–**h** Wild-type HeLa cells or HeLa cells stably expressing the siRNA resistant constructs indicated were mock-transfected or transfected with siRNA targeting endogenous spastin, then fixed and labelled for acetylated tubulin. The percentage of cells developing cellular protrusions was then counted and plotted in **g**, *n* = a minimum of three biological repeats for each experimental condition. Spastin depletion and exogenous spastin expression was validated by immunoblotting (**h**). Note that M1 spastin is present, but in low abundance, in HeLa cells. **i**–**m** Wild-type HeLa cells or HeLa cells stably expressing the siRNA resistant constructs indicated were mock-transfected or transfected with siRNA targeting endogenous spastin, then fixed and labelled for acetylated tubulin. The percentage of cells developing cellular protrusions was then counted and plotted in **l**, *n* = 6 biological repeats. Spastin depletion and exogenous spastin expression was validated by immunoblotting (**m**). *P* values were generated by one-way ANOVA with Tukey’s post hoc test for multiple comparisons

Mammalian cell types express at least two spastin isoforms, a less-abundant full-length form (M1-spastin) and a more highly expressed isoform (M87-spastin) that lacks the N-terminal 86 amino acids of the full-length protein (Fig. 2a) [29]. M1-spastin localises to the ER via a predicted hairpin membrane domain that is specific to this isoform [23, 30]. Here, it participates with the endosomal ESCRT-III protein IST1 in ER–endosome contacts that promote endosomal tubule fission [17]. This M1 isoform (but not the M87 isoform) interacts with protrudin and several ER-resident proteins that have been implicated in HSP, very likely via hairpin membrane domain interactions [30–34]. Although M87-spastin is predominantly cytosolic at steady state, it can also be recruited to endosomes (and presumably ER–endosome contact sites) by ESCRT-III interactions [23, 35].

SiRNA-rescue experiments in HeLa cells have shown that either isoform is sufficient to rescue endosomal tubule fission defects in cells lacking endogenous spastin [15, 17]. Consistent with this, the cellular protrusion phenotype was rescued by either M1- or M87-spastin (Fig. 2b–d, g, h). In contrast, and consistent with the MEF data above, a form of M87-spastin containing the ATPase-deficient disease-associated mutant K388R was unable to rescue the protrusion phenotype (Fig. 2a, e, g, h). This mutant is able to bind but not hydrolyse ATP and so is unable to modify microtubules, nor is it able to promote efficient endosomal tubule fission [13, 15]. Spastin binds to ESCRT-III-associated proteins via interactions with its microtubule interacting and trafficking (MIT) domain (Fig. 2a), and introduction of the MIT F124D mutation, which both blocks these interactions and fails to support endosomal tubule fission, similarly caused failure to rescue protrusion formation (Fig. 2f–h) [36]. Finally, a form of M87 spastin lacking a region that is necessary and sufficient for microtubule binding (myc-M87-spastin∆MTB, Fig. 2a) was also unable to rescue protrusion formation (Fig. 2i–m) [37]. We concluded that either spastin isoform is sufficient to inhibit protrusion formation, although neither was specifically necessary, and that this property of spastin requires its ability to bind microtubules, hydrolyse ATP (and so modify microtubules), and to interact with ESCRT-III. Thus, the properties of spastin that are required to prevent protrusions are the same as those previously identified as being required for it to promote endosomal tubule fission [15, 17].

### Spastin-binding ESCRT-III-associated proteins inhibit protrusion formation

**Fig. 3:**
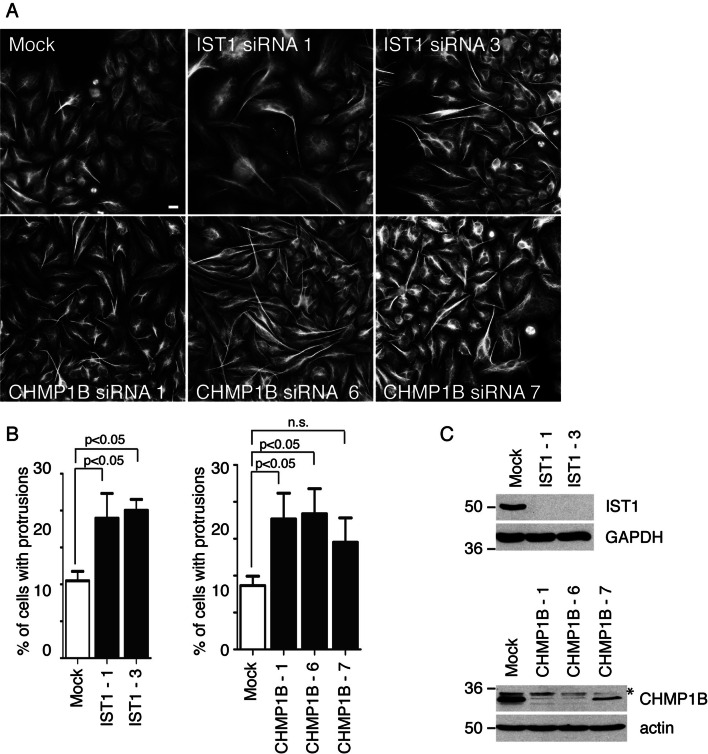
IST1 and CHMP1B prevent protrusion formation. **a** HeLa cells were subject to mock transfection or transfection with the siRNA oligonucleotides indicated, then labelled with acetylated tubulin and the percentage of cells showing protrusions quantified. The mean result of six such experiments for each protein depleted is shown in **b**. Protein depletion was validated by immunoblotting (**c**). The higher molecular weight band (asterisk) recognised by the CHMP1B antibody represents a non-specific cross-reaction. *P* values were generated by one-way ANOVA with Dunnett’s post hoc test for multiple comparisons. Scale bar = 20 μm

The spastin MIT domain is able to interact with two ESCRT-III-associated proteins, IST1 and CHMP1B [35, 36, 38]. As spastin required the capacity to interact with ESCRT-III to suppress protrusion formation, we tested the effect of cellular depletion of these proteins and found that lack of either protein caused cellular protrusions to develop (Fig. 3a–c).

We showed previously that IST1 is involved with spastin in regulation of endosomal tubule fission [15, 17]. This caused cells lacking IST1 to develop increased numbers of SNX1-positive endosomal tubules. As IST1 and CHMP1B have been proposed to act together as a tubule fission machinery, somewhat surprisingly in this previous work, we found no significant effect of CHMP1B depletion on endosomal tubule numbers [39]. We revisited this using a more sensitive assay, reporting the percentage of cells with at least one long endosomal tubule, and found that cells lacking CHMP1B also showed an increase in endosomal tubulation (Supplementary Figure 2). Thus, the effects of spastin, IST1 and CHMP1B depletion in inhibiting endosomal tubule fission mirror their effects on inhibiting protrusion formation.

### Protrusion formation in cells lacking spastin requires protrudin and KIF5A

**Fig. 4:**
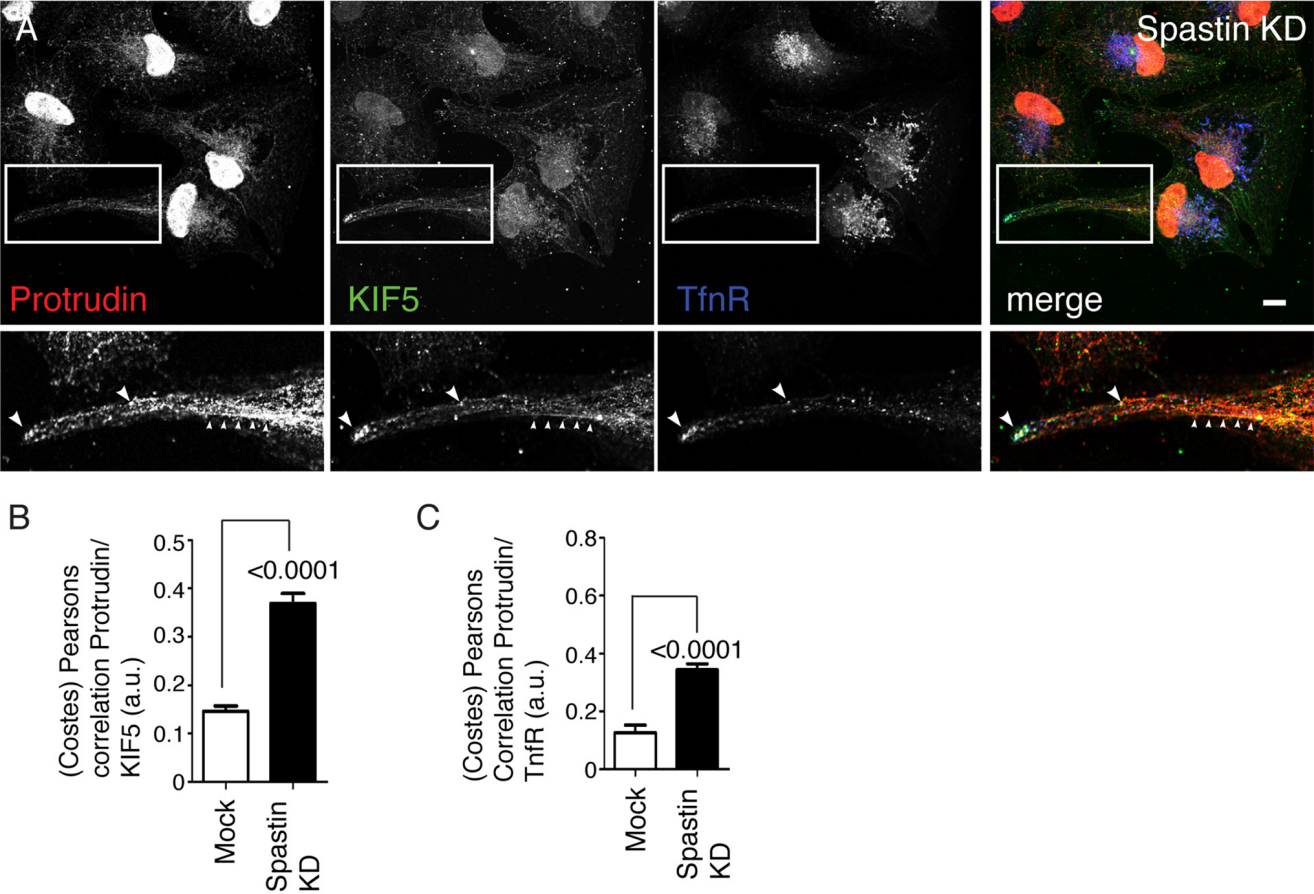
Increased co-localisation between protrudin, KIF5 and endosomal markers in cells lacking spastin. **a** Hela cells were transfected with spastin siRNA, then treated with a saponin-based buffer to wash out soluble cytosolic signal, before being fixed and visualised by immunofluorescence microscopy versus the antibodies shown. The lower panels show higher magnification views of the boxed areas in the upper panels. Large arrowheads indicate examples of co-localised puncta, small arrowheads indicate co-localisation of KIF5 and protrudin along a linear structure. **b**, **c** Cells were labelled with antibodies to the proteins indicated, then the correlation between marker signals was quantified in mock-transfected cells or cells depleted of spastin by siRNA transfection. **b***n* = 34 cell in each condition, **c***n* = 10 cells mock and 17 cells spastin KD. Scale bar = 20 μm. *P* values were generated by unpaired *t* test

We examined whether the protrusions seen in cells lacking spastin were generated by the protrudin-KIF5-dependent mechanism proposed by Raiborg et al. [19]. We began by examining whether the subcellular localization of protrudin and KIF5 was affected by spastin depletion. Protrudin and KIF5 were both present in protrusions that developed in HeLa cells lacking spastin (Fig. 4a; Supplementary Figure 3). KIF5 and protrudin showed minimal co-localisation in wild-type cells, but punctate co-localisation was observed in cells lacking spastin (Fig. 4a, b; Supplementary Figure 3). Co-localised KIF5 and protrudin puncta were often arranged in linear arrays within the protrusions, probably representing alignment along a microtubule, and in some cases co-localised with an endosomal marker (Fig. 4a, c; Supplementary Figure 3). Thus, spastin depletion promotes co-localisation of protrudin and KIF5A in cellular protrusions.

**Fig. 5:**
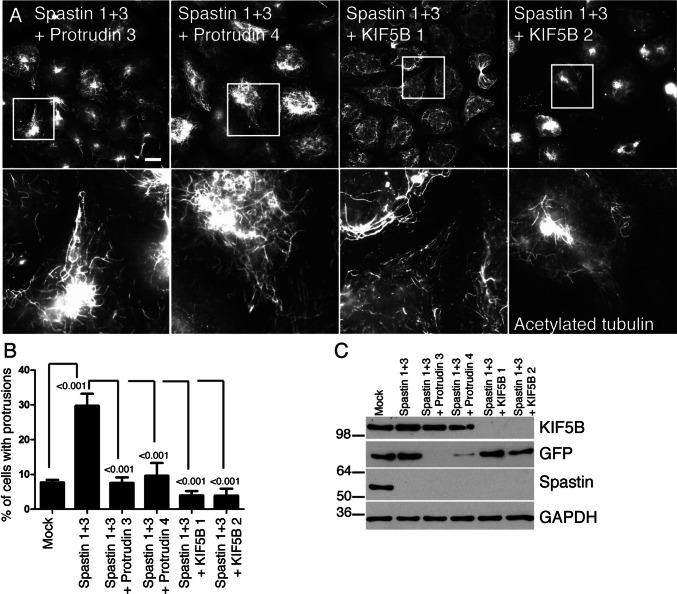
Protrudin and KIF5 are required for the protrusions that develop in cells lacking spastin. **a**–**c** HeLa cells were mock-transfected, or transfected with the indicated spastin siRNAs, spastin and protrudin siRNAs, or spastin and KIF5B siRNAs, then labelled for acetylated tubulin. Selected representative images are shown in **a**, with the lower panels being higher magnification views of the boxed areas shown in the upper panels. The percentage of cells with protrusions was quantified in **b**, *n* = at least 4 biological repeats for each experimental condition. Protein depletion was confirmed by immunoblotting (**c**). In these experiments, as an antibody to endogenous protrudin was not then available, protrudin depletion was verified by carrying out siRNA transfection experiments in parallel, in cells transiently expressing GFP-protrudin. Anti-GFP immunoblotting of these cells is shown. Subsequent validation that the siRNAs used could deplete cells of endogenous protrudin is shown in Supplementary Figure 4. *P* values generated by one-way ANOVA with Tukey’s post hoc test for multiple comparisons. Scale bar = 20 μm

We then analysed whether protrudin or KIF5 are required for the protrusion formation in cells lacking spastin. Using double siRNA knock down experiments in HeLa cells, we found that the spastin protrusion phenotype was efficiently rescued by concurrent knock-down of KIF5B or protrudin, using two independent siRNAs to target each transcript (Fig. 5a–c; Supplementary Figure 4).

### Spastin controls the polarised traffic of membrane receptors implicated in HSP

Having shown that cells lacking spastin develop a polarised morphology, we examined whether this affected polarisation of cell surface receptors. Upregulated Bone Morphogenetic Protein (BMP) signalling, caused by altered endosomal or secretory pathway trafficking of BMP receptors (BMPRs), has been proposed as a potential unifying mechanism in several subtypes of HSP [3, 22]. With specific reference to spastin, zebrafish lacking the M1-spastin isoform have motor axonal mistargeting that is caused by upregulated BMP signalling [40], and we have shown that HeLa cells lacking the protein have upregulated BMP signalling, without alteration in the total cellular amount of the BMP receptor BMPRII [22]. We, therefore, examined whether this upregulated BMP signalling might be associated with a more polarised distribution of BMP receptors.

**Fig. 6:**
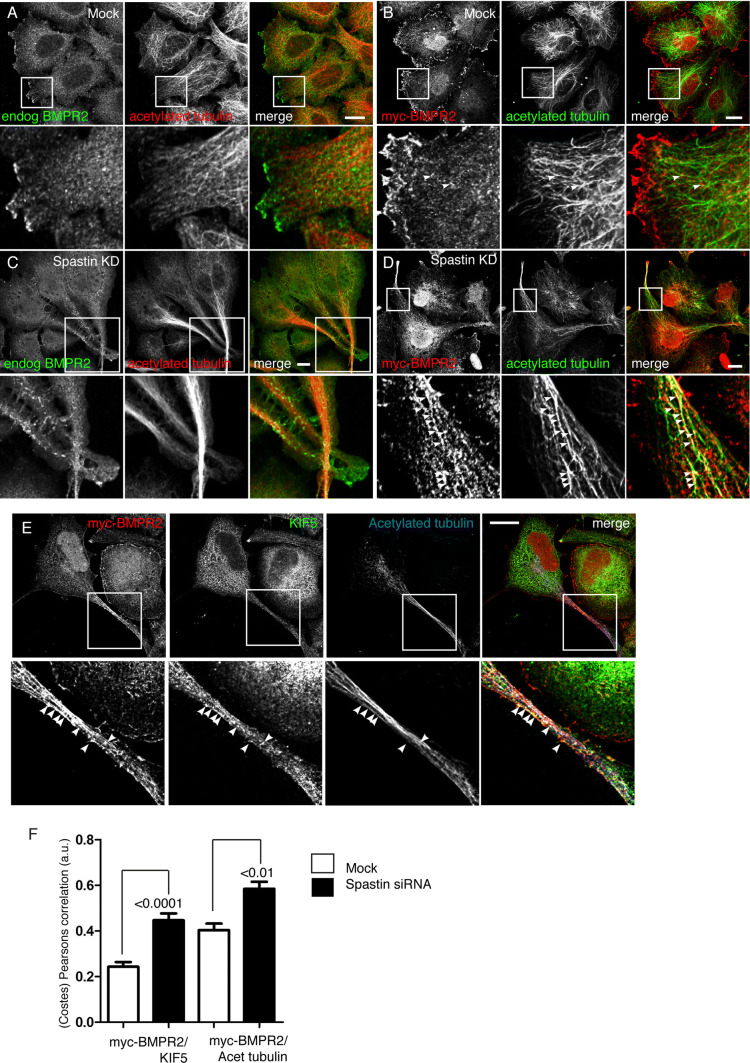
Spastin depletion causes polarised distribution of BMPRII. **a**–**d** Wild-type HeLa cells (**a**, **c**) or HeLa cells staby expressing myc-BMPRII (**b**, **d**) were mock-transfected (**a**, **b**) or subject to spastin KD by siRNA transfection (**c**, **d**), then labelled for the proteins indicated. The panels in the second and fourth rows show higher magnification views of the boxed regions in the panels above and arrows show regions of colocalisation. **e** HeLa cells stably expressing myc-BMPRII were subject to spastin KD by siRNA transfection, then labelled for the proteins indicated. The lower panels are higher magnification views of the boxed protrusion region in the upper panels. Arrows indicate regions of co-localisation. **f** Colocalisation between BMPRII versus KIF5 or acetylated tubulin was measured in HeLa cells stably expressing myc-BMPRII and treated with mock siRNA transfection, or spastin siRNA transfection. At least 13 cells quantified in each condition. *P* values generated by unpaired *t* tests. Scale bars = 20 μm

At steady state in wild-type cells, epitope-tagged BMPRII is present at the plasma membrane and in early and recycling endosomes [22]. Consistent with this, in wild-type HeLa cells both endogenous BMPRII and stably expressed myc-tagged BMPRII were present at the plasma membrane in a non-polarised distribution, and also in intracellular vesicles that showed occasional alignment with microtubules (Fig. 6a, b). However, in cells lacking spastin, both endogenous and stably expressed BMPRII became much more polarised, with strong signal being present on the plasma membrane of protrusions, especially at their tip (Fig. 6c, d). BMPRII signal was also present intracellularly within the protrusions, where it aligned with markers of microtubules and KIF5. Although we cannot completely exclude this overlap as being the result of the concentration of the markers within a confined cellular space, it is consistent with the idea that BMPR-containing endosomes are subject to KIF5-dependent microtubule-based transport into the protrusions (Fig. 6e, f). In addition, in cells lacking spastin, there was generally increased colocalisation between endogenous BMPRII and protrudin (Supplementary Figure 5).

**Fig. 7:**
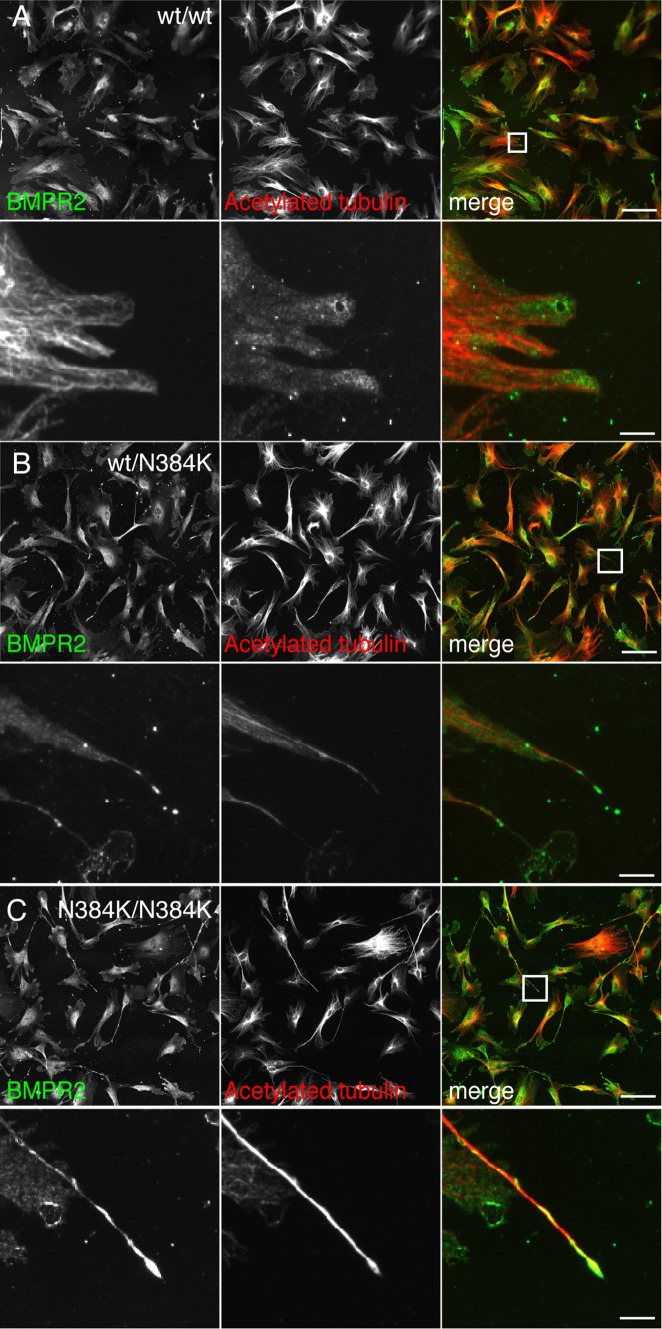
BMPRII shows a polarised distribution in primary cells from spastin^N384K/N384K^ mice. **a** MEFs from mice with the genotypes indicated were fixed and labelled for endogenous BMPRII and acetylated tubulin. The second, fourth and sixth panels show higher magnification views of the boxed areas indicated in the panels above. BMPRII concentrates at the tip of the cellular protrusions that develop in cells from the spastin^N384K/N384K^ mice. Scale bars in rows 1, 3 and 5 = 100 μm, in 2, 4 and 6 = 20 μm

We confirmed the physiological relevance of these observations in MEFs from spastin^N384K/N384K^ mice, where we found that endogenous BMPRII had a highly polarised distribution at the tips of the protrusions that developed in heterozygous, and especially homozygous, knock-in mice (Fig. 7).

## Discussion

A model for protrudin-dependent protrusion formation has been proposed, in which ER-localised protrudin interacts with late endosomes by co-incident detection of RAB7 and phosphatidylinositol 3-phosphate. These contacts facilitate transfer of KIF5 that has been captured by protrudin, to the late endosomal motor adaptor FYCO1. This process promotes microtubule-motor-dependent movement of late endosomes towards the plasma membrane, with which they fuse, thereby delivering membrane to drive protrusion formation [19]. Our work now demonstrates that spastin regulates this process. HeLa cells lacking spastin developed long protrusions that required protrudin and KIF5 for their formation. Cells lacking spastin had increased co-localisation of protrudin, KIF5 and endosomal markers within protrusions, consistent with previous observations that over-expression of protrudin increases its co-localisation with FYCO1-bearing endosomes [19]. Considered together, our results indicate that spastin inhibits protrudin-dependent protrusion formation. This role for spastin is physiologically relevant, as embryonic fibroblasts from a spastin knock-in mouse model, which expresses at endogenous levels an ATPase-defective form of the protein, also became polarised and developed long cellular protrusions.

**Fig. 8:**
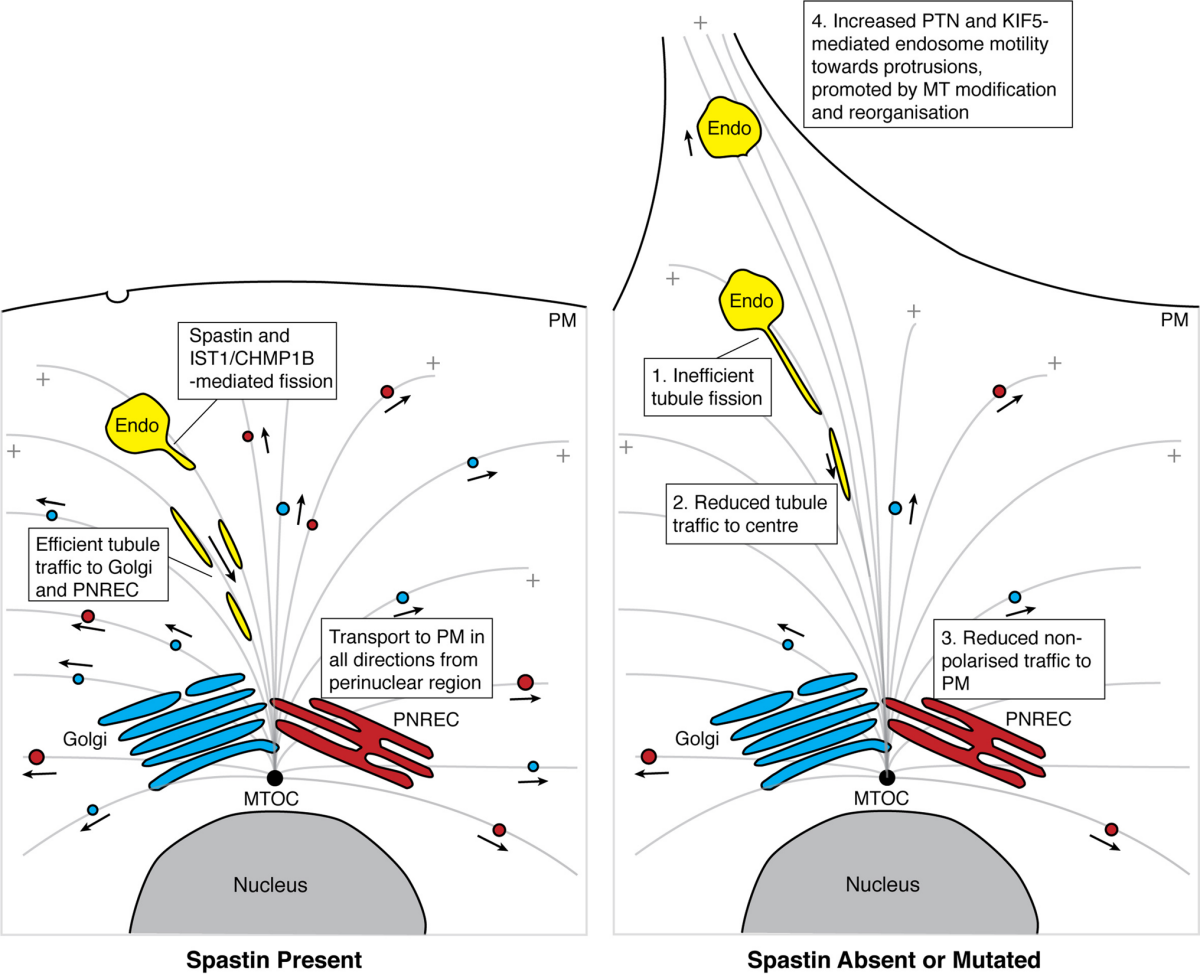
Schematic diagram of possible roles of spastin in generalised versus polarised membrane traffic. The cartoon on the left shows membrane traffic pathways in a cell without a protrusion, in the presence of functional spastin. Membrane that traffics to the cell centre in endosomal tubules (a process that requires spastin [15, 17]) can be redistributed from there to anywhere on the plasma membrane (PM), i.e., in a non-polarised fashion, because of the close localisation of the Golgi apparatus and perinuclear recycling compartment (PNREC) to the microtubule organising centre (MTOC). Endosomes are held in position by a balance between the activity of plus-end (+) and minus-end-directed motor proteins. The cartoon on the right shows the effects of absence of functional spastin, and how this might lead to protrusion formation. Firstly, this causes inefficient endosomal tubule fission (1). This would be expected to reduce membrane delivery to the Golgi apparatus or PNREC (2), with consequent reductions in non-polarised, generalised membrane delivery to the plasma membrane (3). Thus, spastin would tend to switch off generalised recycling. In cells lacking spastin, the protrusions are protrudin (PTN) and KIF5-dependent, implying increased KIF5-dependent traffic of endosomes towards protrusions [19]. This may be aided by microtubule alterations caused by lack of spastin, which could promote long-range plus-end-directed transport or inhibit dynein-dependent minus-end-directed transport of endosomes or endosomal tubules (4). Endosomal compartments are shown in yellow, the Golgi apparatus and post-Golgi carriers in blue, the PNREC and post-PNREC carriers in red

How could spastin antagonise the function of protrudin? Spastin promotes endosomal tubule fission of SNX1 and SNX4 tubules, and the functional properties required for spastin to regulate tubule fission are the same as those required to suppress protrusion formation, hinting that the two processes may be related [15]. Both SNX1 and SNX4 tubules move along microtubules predominantly to the cell centre [15, 41]. From here, membrane may be delivered in a non-polarised fashion to the plasma membrane, either via the Golgi apparatus or perinuclear recycling endosomes. Thus, by inhibiting this process lack of spastin would be expected to inhibit generalised recycling (Fig. 8). In this sense, spastin would be antagonistic to the function of protrudin in generalised recycling, which it has been proposed to inhibit by locking RAB11 in the inactive GDP-bound state. This model would also be consistent with our observation that either spastin isoform can support ER-mediated endosomal tubule fission [15]. We have previously suggested that in the physiological situation, efficient spastin hexamer formation involves nucleation by M1-spastin at ER–endosome contacts involved in tubule fission, followed by recruitment of M87-spastin from a cytosolic pool to form functional spastin hexamers. This capacity of M1 spastin may perhaps be compensated for by direct recruitment of M87-spastin to endosomes by IST1 interaction.

In addition to affecting protrusion formation via effects on endosomal tubule fission, spastin could more directly influence protrudin-dependent microtubule-based polarised endosome motility. ER tubules are closely associated with microtubules [42], and the interaction and co-localisation observed between spastin and protrudin suggest that spastin would be positioned to sever or nick microtubules in close proximity to protrudin. Severing of these microtubules could counteract long-range KIF5-dependent movement of cargo towards the cell periphery, by physically breaking the microtubule rail or by facilitating dynein loading at new microtubule plus ends, so tipping the balance towards non-polarised minus-end-directed transport towards the centrosome. Spastin-induced nicking of microtubules could have a similar effect, as it introduces patches of new GTP-tubulin into the microtubule lattice. In vitro such patches recruit plus-end proteins to the microtubule, which could enhance dynein loading and minus-end transport [6]. We have proposed a similar model of promoting plus-end dynein motor protein loading to explain how spastin drives fission of endosomal tubules from the endosomal body, which is known to involve a pulling force by dynein [15]. Finally, the physiological consequences of spastin-induced microtubule nicking are not yet known, but if this were to lead to alterations in tubulin post-translational modification, it could conceivably have effects on MT-dependent motility of endocytic organelles, as exemplified by the observation that lysosomal transport stalls on patches of detyrosinated tubulin [43]. Interestingly, the protrusions that developed in cells lacking spastin were enriched for acetylated tubulin, a marker of long-lived microtubules that are associated with long-range transport.

It is clear from our work that regulation of spastin’s activity could provide an important cellular switch between generalised and polarised membrane traffic. In this regard, we have also discovered a novel role for the ESCRT-III-associated proteins IST1 and CHMP1B in inhibiting protrusion formation, via their interaction with spastin. Although the steady-state localisation of CHMP1B is not known (due to lack of suitable antibodies), at steady state, IST1 mainly localises to a subdomain of the early sorting endosomal compartment. Thus, as the endosome matures to the RAB7-positive late endosome, removal of IST1 could act as a temporal switch to reduce spastin-mediated endosomal tubule fission, thereby inhibiting generalised recycling and promoting protrudin-dependent endosomal motility. What properties of late endosomes determine whether they are subject to protrudin-dependent anterograde motility or to other anterograde or retrograde motility mechanisms are not fully understood, but at least involve membrane cholesterol, oxysterol or phospholipid composition [44].

As in protrudin over-expressing cells, cells depleted of spastin developed accumulations of endosomal markers at their tips, consistent with the model of increased anterograde KIF5-dependent vesicular traffic within the protrusions. Late endosomes at the tips of protrudin-induced protrusions fuse with the plasma membrane in a synaptotagmin-VIII-dependent fashion, thus providing a mechanism by which membrane receptor distribution could become polarised [19]. We examined whether spastin depletion affected the polarisation of BMP receptors, as this pathway has been implicated in HSP; cellular or in vivo animal models of multiple HSP subtypes have demonstrated upregulated BMP signalling, and axonal abnormalities in vertebrate and invertebrate HSP models have been rescued by inhibiting this upregulated signalling [22, 45–50]. In several cases, these BMP signalling abnormalities have been associated with abnormal endosomal trafficking of the BMP type II receptor or its homologues [22, 46, 49]. HeLa cells lacking spastin have upregulated BMP signalling, but no alteration in the total cellular amount of the BMP receptor BMPRII [22]. However, we now show that spastin influences BMPRII distribution; in HeLa cells lacking spastin, BMPRII signal was concentrated within protrusions, where it co-localised with KIF5, and also on the plasma membrane surrounding the protrusions. The physiological relevance of this role of spastin in regulating BMPRII distribution was confirmed in primary embryonic fibroblasts derived from spastin^N384K/N384K^ mice, where BMPRII signal was also concentrated at the tips of protrusions. Thus, the protrusion phenotype associated with loss or dysfunction of spastin causes polarisation of a pathologically relevant membrane receptor. It will be interesting in the future to determine how spastin influences the role of protrudin in neurons, and the relevance of this function of the protein to the pathogenesis of HSP. This would involve thorough characterisation of neuronal and axonal phenotypes in neurons lacking each or both proteins, as well as analysis of the corresponding effects on polarised endosomal membrane traffic, including of key receptors implicated in HSP such as BMPRII.

In summary, we have identified a physiologically relevant role for spastin in controlling the balance between generalised and polarised recycling. In addition, by showing that IST1 and CHMP1B are involved in this process, our work adds to the list of biological functions in which the ESCRT complexes have been implicated, which includes intralumenal vesicle formation at the late endosome, cytokinesis, nuclear envelope reformation, exocytosis and viral budding amongst others [51].

## Electronic supplementary material

Below is the link to the electronic supplementary material.

Supplementary Figure 1. Further characterisation of protrusions. Hela cells depleted of spastin by siRNA knock-down (A) or MEFS form spastin^N384K/N384K^ mice (B) were fixed, labelled with acetylated tubulin, the actin stain phalloidin, and the whole cell stain HCS CellMask (Thermo Fisher), then visualised by confocal microscopy. Examples of protrusions labelled for all three markers are highlighted with arrowheads. C) MRC5 cells were mock-transfected or subject to spastin knock-down with the oligonucleotides indicated, then labelled with for acetylated tubulin. The mean percentage of cells with protrusions was counted in n=4 biological repeats and plotted in (D). Spastin protein depletion was verified by immunoblotting (E). P-values generated by paired *t* test. Scale bars = 20 μm. (JPEG 1247 kb)

Supplementary Figure 2. CHMP1B regulates endosomal tubulation. A) HeLa cells were subjected to mock transfection or transfected with the CHMP1B siRNA oligonucleotides indicated, then fixed and labelled with an antibody to endogenous SNX1, a marker of endosomal tubules. The percentage of cells with at least one endosomal tubule longer than 2 μm was counted (100 cells per condition) and the mean results of 7 such experiments are plotted in B). P-values were generated by one-way ANOVA with Dunnett’s post hoc test for multiple comparisons. Scale bars= 10 μm in large panels, 2 μm in insets, which show a higher magnification view of the boxed areas indicated in the large images. (JPEG 335 kb)

Supplementary Figure 3. Polarisation of markers following spastin depletion. A) HeLa cells were subjected to mock transfection or transfected with spastin siRNA, and labelled with the antibodies indicated. The inset boxes show higher magnification view of the boxed areas. B) HeLa cells were subjected to mock transfection or transfected with spastin siRNA, and labelled with the antibodies indicated. The bottom panel shows a higher magnification view of the boxed area in the panel above it. Arrowheads indicate co-localised puncta. C) HeLa cells were subjected to mock transfection or transfected with protrudin siRNA, then fixed and labelled using an antibody to endogenous protrudin. Scale bar=10 μm. (JPEG 2039 kb)

Supplementary Figure 4. Specificity of the protrudin antibody for immunoblotting and validation of efficiency of protrudin siRNA. HeLa cells were subjected to mock transfection or transfected with the spastin or protrudin siRNAs indicated, then the cells were lysed and immunoblotted versus an antibody to endogenous protrudin, to confirm protein depletion. GAPDH blotting serves to validate equal protein loading in each lane. (JPEG 122 kb)

Supplementary Figure 5. Increased co-localisation between protrudin and BMPR2 in cells lacking spastin. HeLa cells were subjected to mock transfection or transfected with spastin siRNA, and labelled with the antibodies indicated. The inset boxes show higher magnification view of the boxed areas. Arrows indicate co-localised puncta. Protrudin and BMPR2 co-localisation was quantified by Pearson’s correlation and the results from 10 cells in each condition are plotted in (B). Bars show means +/- standard deviation. P-value generated by unpaired *t* test. Scale bars=10 μm. (JPEG 1016 kb)
