# Supplementary figures and images for: ESCRT-III-associated proteins and spastin inhibit protrudin-dependent polarised membrane traffic
Source: Cell Mol Life Sci. 2019 Oct 5;77(13):2641–58. doi: 10.1007/s00018-019-03313-z (PMC7320071; doi:10.1007/s00018-019-03313-z)

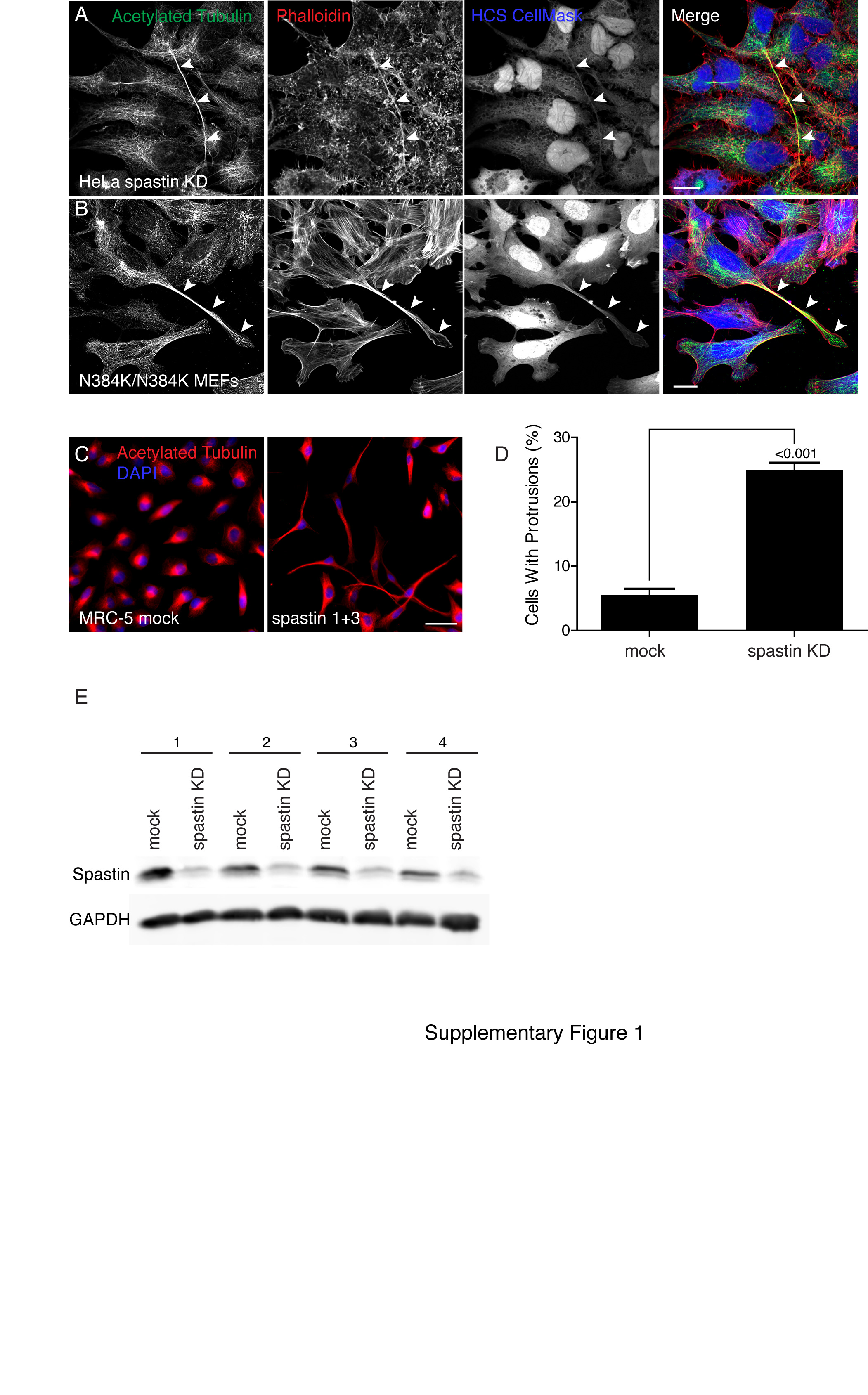

Supplement: Supplementary file 1 — Supplementary Figure 1. Further characterisation of protrusions. Hela cells depleted of spastin by siRNA knock-down (A) or MEFS form spastinN384K/N384K mice (B) were fixed, labelled with acetylated tubulin, the actin stain phalloidin, and the whole cell stain HCS CellMask (Thermo Fisher), then visualised by confocal microscopy. Examples of protrusions labelled for all three markers are highlighted with arrowheads. C) MRC5 cells were mock-transfected or subject to spastin knock-down with the oligonucleotides indicated, then labelled with for acetylated tubulin. The mean percentage of cells with protrusions was counted in n=4 biological repeats and plotted in (D). Spastin protein depletion was verified by immunoblotting (E). P-values generated by paired t test. Scale bars = 20 μm. (JPEG 1247 kb) [file 18_2019_3313_MOESM1_ESM.jpg]

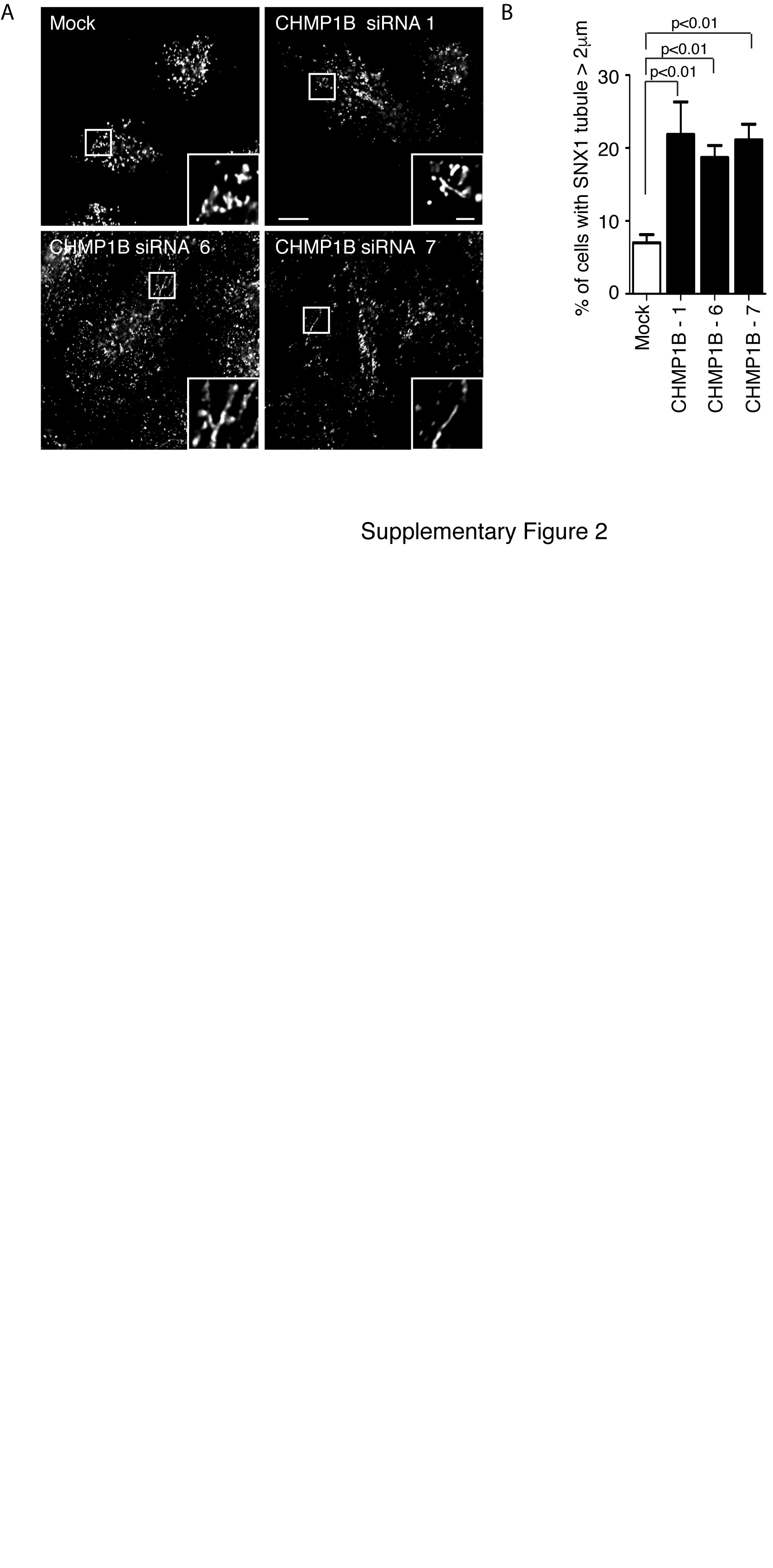

Supplement: Supplementary file 2 — Supplementary Figure 2. CHMP1B regulates endosomal tubulation. A) HeLa cells were subjected to mock transfection or transfected with the CHMP1B siRNA oligonucleotides indicated, then fixed and labelled with an antibody to endogenous SNX1, a marker of endosomal tubules. The percentage of cells with at least one endosomal tubule longer than 2 μm was counted (100 cells per condition) and the mean results of 7 such experiments are plotted in B). P-values were generated by one-way ANOVA with Dunnett’s post hoc test for multiple comparisons. Scale bars= 10 μm in large panels, 2 μm in insets, which show a higher magnification view of the boxed areas indicated in the large images. (JPEG 335 kb) [file 18_2019_3313_MOESM2_ESM.jpg]

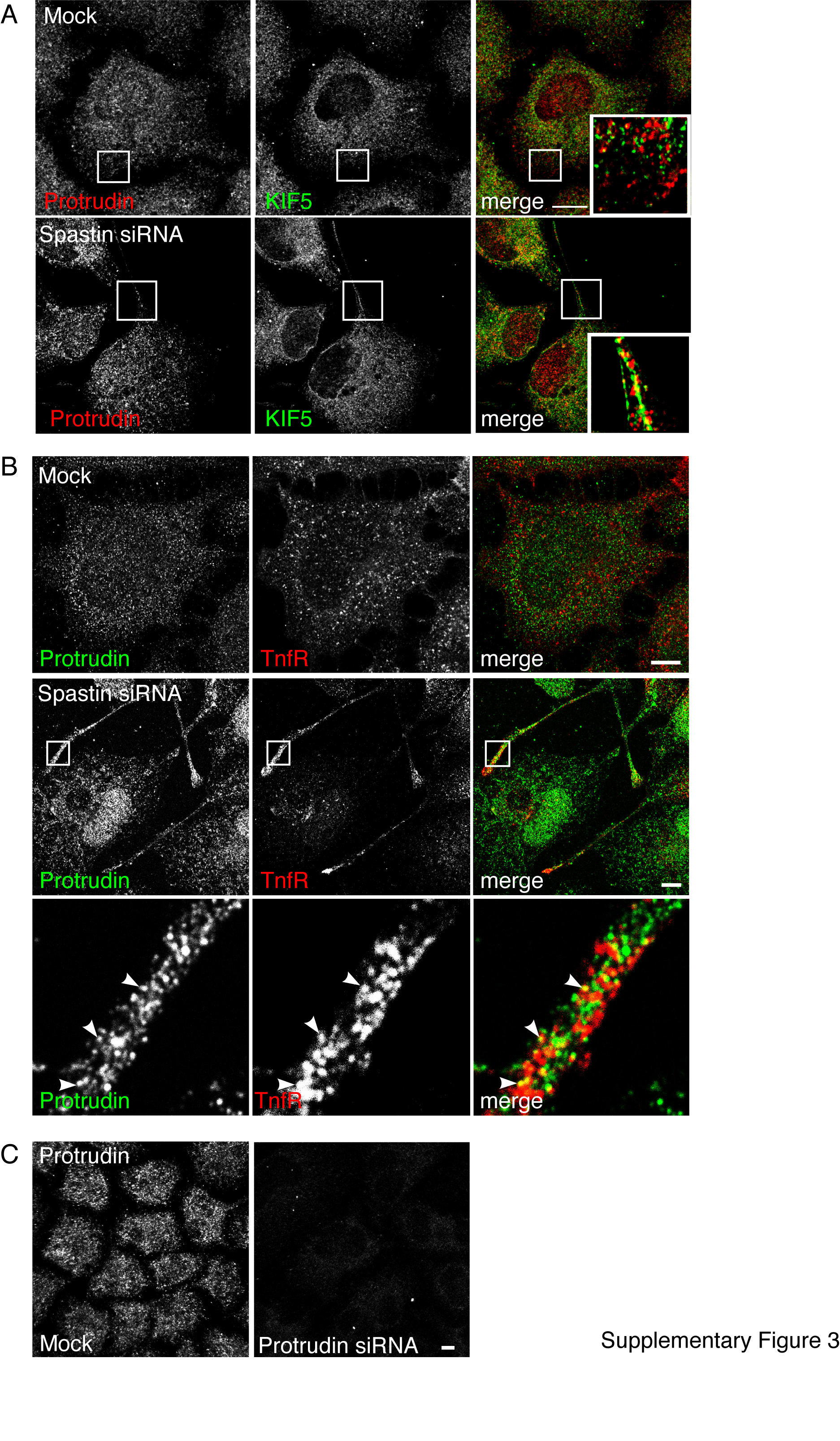

Supplement: Supplementary file 3 — Supplementary Figure 3. Polarisation of markers following spastin depletion. A) HeLa cells were subjected to mock transfection or transfected with spastin siRNA, and labelled with the antibodies indicated. The inset boxes show higher magnification view of the boxed areas. B) HeLa cells were subjected to mock transfection or transfected with spastin siRNA, and labelled with the antibodies indicated. The bottom panel shows a higher magnification view of the boxed area in the panel above it. Arrowheads indicate co-localised puncta. C) HeLa cells were subjected to mock transfection or transfected with protrudin siRNA, then fixed and labelled using an antibody to endogenous protrudin. Scale bar=10 μm. (JPEG 2039 kb) [file 18_2019_3313_MOESM3_ESM.jpg]

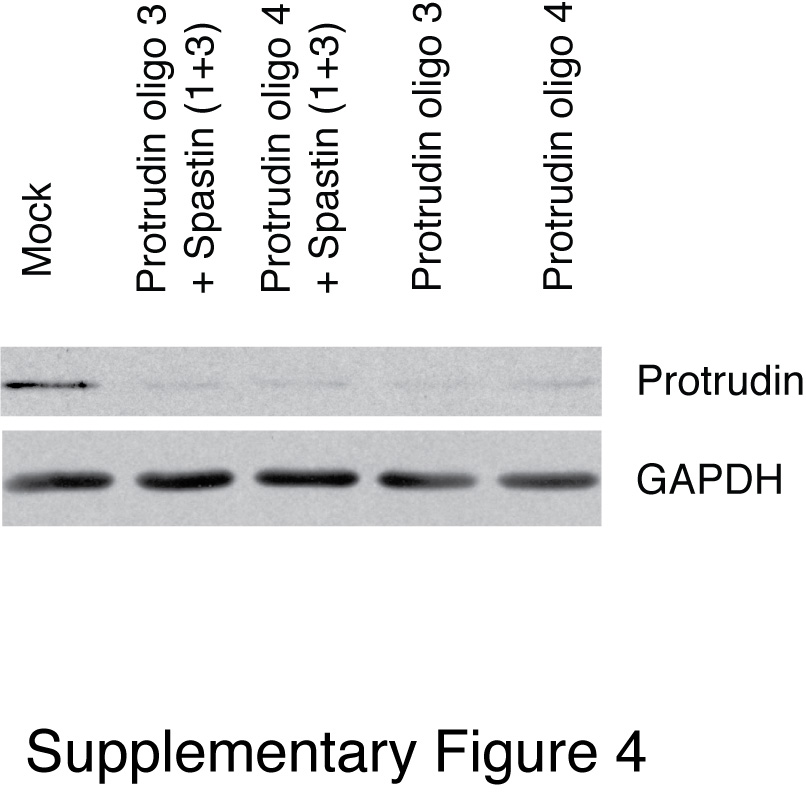

Supplement: Supplementary file 4 — Supplementary Figure 4. Specificity of the protrudin antibody for immunoblotting and validation of efficiency of protrudin siRNA. HeLa cells were subjected to mock transfection or transfected with the spastin or protrudin siRNAs indicated, then the cells were lysed and immunoblotted versus an antibody to endogenous protrudin, to confirm protein depletion. GAPDH blotting serves to validate equal protein loading in each lane. (JPEG 122 kb) [file 18_2019_3313_MOESM4_ESM.jpg]

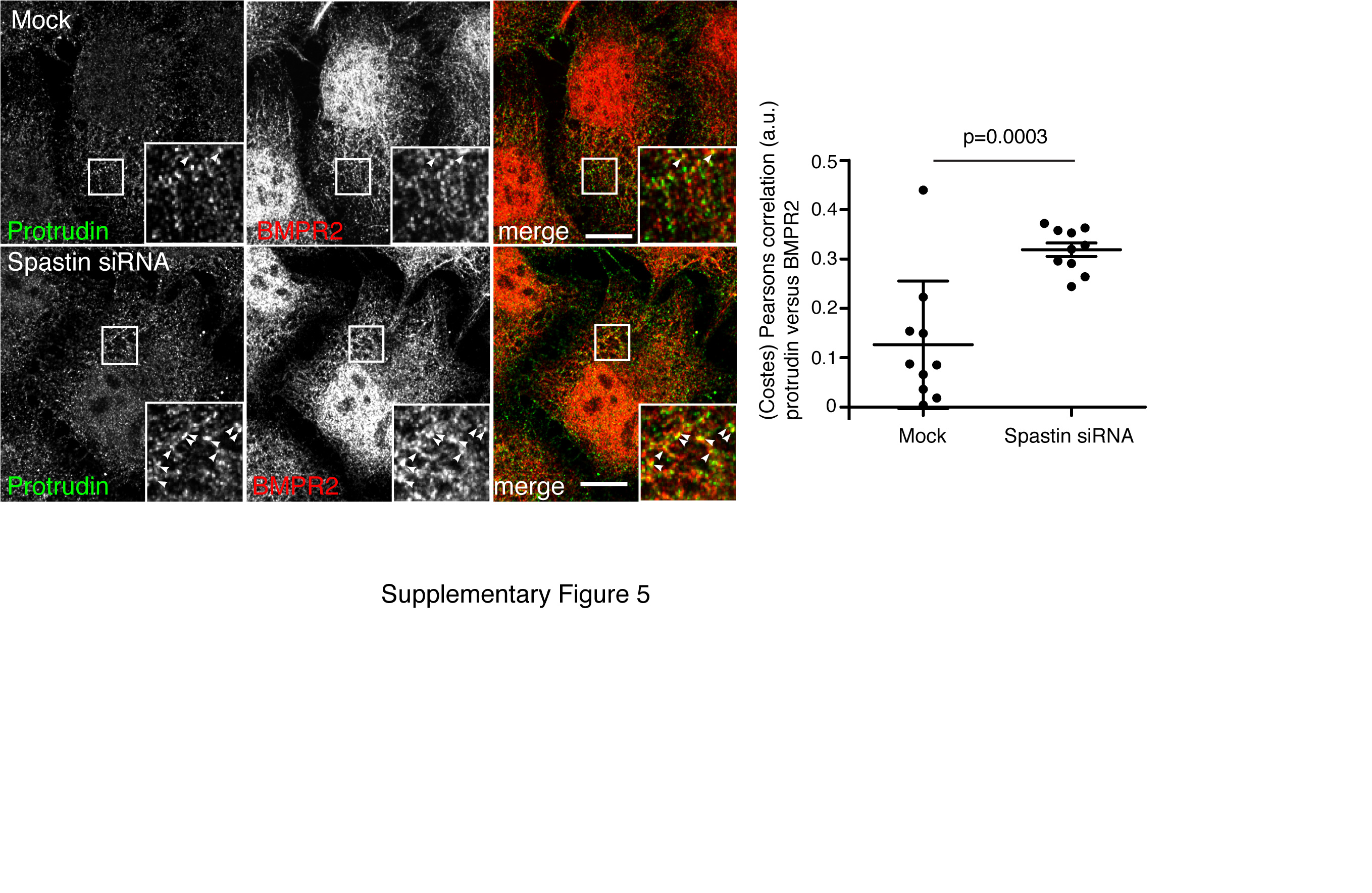

Supplement: Supplementary file 5 — Supplementary Figure 5. Increased co-localisation between protrudin and BMPR2 in cells lacking spastin. HeLa cells were subjected to mock transfection or transfected with spastin siRNA, and labelled with the antibodies indicated. The inset boxes show higher magnification view of the boxed areas. Arrows indicate co-localised puncta. Protrudin and BMPR2 co-localisation was quantified by Pearson’s correlation and the results from 10 cells in each condition are plotted in (B). Bars show means +/- standard deviation. P-value generated by unpaired t test. Scale bars=10 μm. (JPEG 1016 kb) [file 18_2019_3313_MOESM5_ESM.jpg]
